# Sonodynamic Therapy Using Protoporphyrin IX Conjugated to Gold Nanoparticles: An *In Vivo *Study on a Colon Tumor Model

**Published:** 2012

**Authors:** Ahmad Shanei, Ameneh Sazgarnia, Naser Tayyebi Meibodi, Hossein Eshghi, Mohammad Hassanzadeh-Khayyat, Habibollah Esmaily, Neda Attaran Kakhki

**Affiliations:** 1 *Department of Medical Physics and Medical Engineering, School of Medicine, Isfahan University of Medical Sciences, Isfahan, Iran*; 2 *Research Centre and Department of Medical Physics, School of Medicine, Mashhad University of Medical Sciences, Mashhad, Iran*; 3 *Skin Research Centre and Department of Pathology, Emam Reza Hospital, Mashhad University of Medical Sciences, Mashhad, Iran*; 4 *Department of Chemistry, School of Science, Ferdowsi University of Mashhad, Mashhad, Iran*; 5 *Pharmaceutical Research Centre, School of Pharmacy, Mashhad University of Medical Sciences, Vakilabad Blvd., School of Pharmacy, Mashhad, Iran*; 6 *Department ofCommunity Medicine &Public Health, School of Health, Mashhad University of Medical Sciences, Mashhad, Iran*

**Keywords:** Acoustic, Cavitation, Nanoparticle, Protoporphyrin IX, Ultrasound

## Abstract

**Objective(s):**

Sonodynamic therapy is a physical treatment which utilizes ultrasound waves with an appropriate sensitizer such as protoporphyrin IX (PpIX). The activation of sensitizer depends on cavitation, and therefore, high intensity ultrasound is an important necessity. Beside, high intensity ultrasound can induce side effects on the healthy tissues which have surrounded tumor. The particles in a liquid decrease the ultrasonic intensity threshold needed for onset of cavitation. The non-radiative relaxation time of PpIX in the presence of gold nanoparticles (GNP) is longer than the similar time without GNP.

**Materials and Methods:**

This study was conducted on colon carcinoma tumor in BALB/c mice. The tumors were induced by subcutaneous injection of CT26 cells. Ultrasound irradiation were performed on tumors 24 hr after the injection of PpIX into GNPs. Antitumor effects were estimated by measuring tumor relative volume, doubling time and time being five times of the tumors and by calculating the average survival time of tumor-bearing mice after treatment.

**Results:**

There is no inhibitory effect in control group. Ultrasound irradiation alone showed a slight antitumor effect which was enhanced by ultrasound plus PpIX (SDT). The synergistic inhibitory effect was significant when ultrasound plus PpIX was conjugated to GNPs.

**Conclusion:**

Our experiments suggested a significant synergistic effect of ultrasound combined with Au-PpIX that reduced tumor relative volume and increased average animal survival fraction. This effect was obviously stronger than ultrasound alone and synergistic effect of ultrasound combined with PpIX.

## Introduction

Many clinical methods in support of cancer treatment do not operate as targeted in the tumor. Furthermore, the researchers are looking for a good approach to treat cancer which focuses on the tumor area, and induces minimal damage to healthy tissues (1).

One of non-invasive therapeutic applications of ultrasound that has been considered in recent decades is sonodynamic therapy (SDT) (2, 3). 

 SDT provides an enhancement on cytotoxic activities of sonosensitizers in tumor cells by exposure to ultrasound. Ultrasound can focus in a small region and deeply penetrate tissue, thus, SDT may be a useful tool for the clinical treatment of tumors located deep in the body (4). 

Protoporphyrin IX (PpIX) is an efficient hydrophobic sensitizer that is activated by both light and ultrasound waves (5, 6). The subsequent interaction of activated PpIX with molecular oxygen produces cytotoxic reactive oxygen species (ROS), particularly singlet oxygen (^1^O_2_), that causes irreversible destruction of the target tissue (7).

Use of high intensity ultrasound is one of the existing challenges in SDT. The activation of sensitizer depends on cavitation process, and therefore, high intensity ultrasound is an important necessity. Beside, high intensity ultrasound can induce side effects on the healthy tissues which have surrounded tumor (8).

The existence of particle in a liquid provides a nucleation site for cavitation bubble due to its surface roughness, leading to the decrease in the cavitation threshold responsible for the increase in the quantity of bubbles, when the liquid is irradiated by ultrasound (9). Thus, in this context, one approach is based on providing the nucleation sites for the purpose of participating in the formation of cavities in order to reduce the threshold intensity needed for cavitation. 

In this study, as a means for reinforcing the effect of ultrasound irradiation and treatment selectivity in the tumor, the use of a new designed sensitizer has been suggested.

Gold nanoparticles (GNPs) represented as a novel nano-material are applied in the field of cancer therapy because of their special optical properties (10). In recent years, GNPs have also been utilized in cancer diagnosis, treatment and drug delivery (11). Molecular bindings to the GNPs such as antibodies, carbohydrates and pharmacologic agents, target cancer cells easily. Non toxicity, good uptake by mammalian cells and antiangiogenesis property of GNP are the most important aspects for its medical applications (11). 

The nonradiative relaxation time of PpIX in the presence of GNPs is longer than the similar time of PpIX without gold nanoparticles (12). This effect can be used in medical diagnostic and therapeutic applications. On the other hand, sonosensitivity and the capability of PpIX in imaging have been proven (12). Thus in this research, the role of conjugated PpIX to GNPs in creating cavitation and sonodynamic antitumor effect were investigated.

## Materials and Methods

***Preparation*** ***and characterization of Au-PpIX***

**Figure1:**
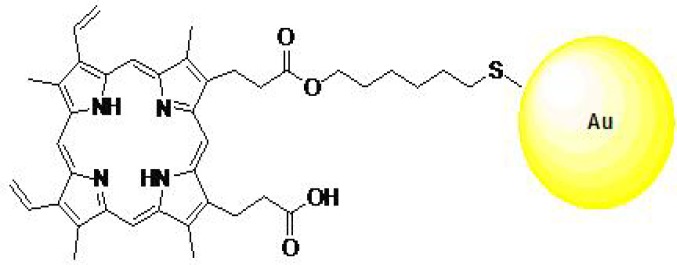
Protoporphyrin conjugated to gold nanoparticle (13)

Protoporphyrin IX () was conjugated to the gold nanoparticles (GNPs) through a bidentate linker. At first, a solution of N, N′-dicyclohexylcarbodiimide (DCC) in CH_2_Cl_2_ was added to a solution of protoporphyrin (PpIX) in DMSO, in order to conjugate protoporphyrin with 6-mercapto-1-hexanol as a linker (PpIX-MH). After 30 min, a solution of 6-mercapto-1- hexanol (MH) in CH_2_Cl_2_ was added to the reaction mixture. Then the final dark-brown solid product was obtained by evaporation. Then a solution of HAuCl_4_.3H_2_O in CH_3_OH was added to a stirred solution of Pp IX-MH in CH_3_OH. After 30 min, a freshly prepared solution of sodium borohydride in CH_3_OH was added to the vigorously stirred reaction mixture. The brown colour of the reaction mixture indicates the formation of GNPs. After 2 hr, the conjugates of GNP with PpIX-MH were separated from methanol by precipitation and purified by successive washing with CH_3_OH and its characterisation was determined (13). Figure 1 shows a structure design of the PpIX-MH-AuNP conjugated gold nanoparticles.

**Figure 2:**
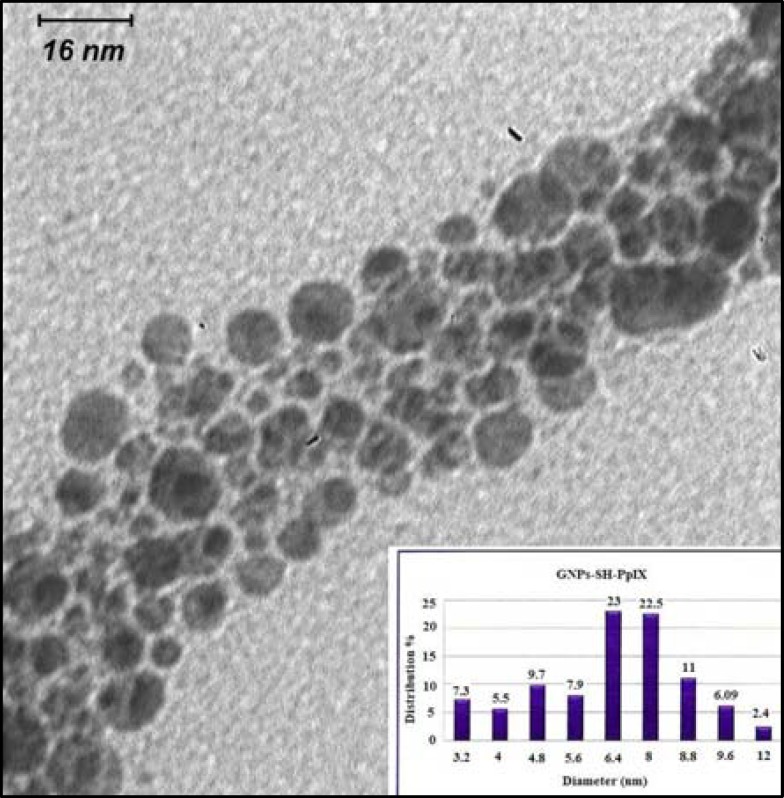
TEM photograph of the PpIX conjugated to gold nanoparticles and histogram for the size distribution of Au-PpIX

**Figure3:**
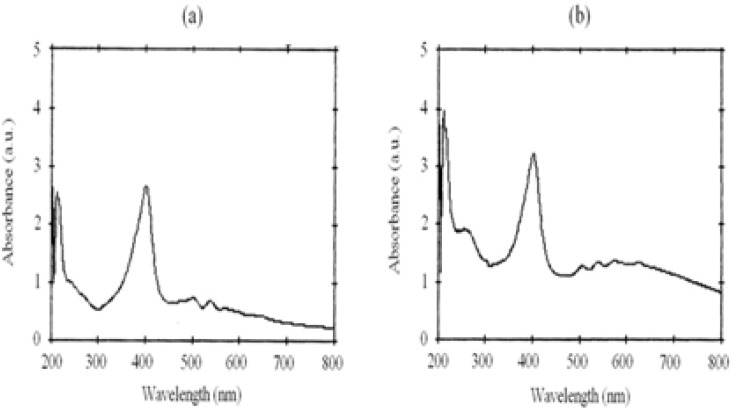
Absorption spectra of (a) PpIX and (b) Au-PpIX in methanol

The characterization of the gold nanoparticles was determined by three techniques: particle size analysis (Zeta sizer, nano-ZS model, Malvern Co., ), UV-visible (UV-vis) spectrophotometery (UV-1700 Phamaspect- Shimadzu) and transmission electron microscopy (LEO 912AB) (14). The transmission electron microscopic (TEM) image of the synthesised PpIX-MH-AuNPs is reported in Figure 2 and Figure 3 shows the UV-visible spectra of the synthesised Pp IX-MH and Pp IX-MH-AuNP conjugated gold nanoparticles.

To determine the conjugating efficiency of the PpIX to GNPs, the fluorescence emission intensity of PpIX conjugated to GNPs were assessed by the fluorimetric method and amount of PpIX was determined. On the other hand, the amount of primary PpIX which has been insinuated through the construction process is also given. So the PpIX concentration conjugated to gold nanoparticles was divided by the primary PpIX concentration and the conjugating efficiency of the PpIX to GNPs was obtained (14). The percentage of conjugated nanogold was also attained by atomic absorption spectrometer as 30.75% PpIX, 11.25% linker and 58% Au in the final conjugated Au-PpIX.

***Cell line and culture conditions***

CT26 cell line derived from a tumor colon carcinoma of a BALB/c mouse were grown in RPMI-1640 supplemented with 10% (v/v) fetal bovine serum (FBS), 50 units/ml Penicillin and 50 μg/ml streptomycin. Cell culture was performed at 37 ^○^C in a 5% CO_2_ humidified incubator. They covered bottom of the flask as a monolayer after 2-3 days of the growth and proliferation of the cells. Exponentially growing cells were trypsinized using 0.05% trypsin-EDTA. The cell survival rate and their number were determined by a hemocytometer using trypan blue (15). 

***Tumor models***

Male BALB/c mice, 6 - 8 weeks weighting on 20-22 g were purchased from Iranian Pasteur Institute. The mice were housed in an animal facility in Medical Physics Research Centre at 23±2 °C, 65% moisture, and an alternative 12 hr darkness and brightness. In order to create a tumor model, CT26 tumor cells (1×10^6 ^cells per mouse) were implanted subcutaneously in the right dorsal animals. When the tumor grew to a volume of about 100±20 mm^3^ approximately 12 days after implantation, the treatment protocols were applied on the tumors (15).

***Animals’ anesthesia***

The mice were anesthetized before the exposure of ultrasound, via intraperitoneal injection of ketamine hydrochloride (60 mg/kg), and xylazine2% (6 mg/kg) (4). 

***Ultrasound generator system***

Irradiation of ultrasound was conducted with a 215A ultrasound generator in continuous mode and frequency of 1.1 MHz with maximum intensity of 2 W/cm^2^ for 3 min (1). Acoustic calibration for the power of the device was carried out in a degassed water tank, using an ultrasound balance power meter (UPM 2000, ) with uncertainty of ± 1mW. All quoted intensities are spatial average temporal average (SATA) in our experiments.

Ultrasound transducer with a surface area of 7.0 cm^2^ was horizontally submerged in the bottom of a glass container filled with degassed water.

***Treatment protocol***

As the tumors volume reached around 100±20 mm^3^, 5 mice were randomly killed, autopsied and subjected to the pathological examinations and affirmed to be a tumor. Then the tumor-bearing mice were randomly divided into 6 groups (each group containing 10 mice): (1) control group, (2) PpIX, (3) Au-PpIX, (4) ultrasound alone, (5) ultrasound plus PpIX and (6) ultrasound plus Au-PpIX. For groups of 2, 5 and 3, 6, PpIX and Au-PpIX were injected into the tumors, respectively. The injective dose was equivalent with 5 mg PpIX / kg weight of mouse (4).

To minimize cavitation events and interference at the tumor surface, tumor region were shaved prior to ultrasound applications using an animal shaver, treated with depilatory cream, washed with liquid soap and rinsed. Then, the naked skin was partially immersed into the water bath and the centre of the tumor was exposed to the ultrasound waves. For all experiments, the cold degassed water was used as ultrasonic medium to avoid thermal effects caused by ultrasound irradiation. For the treatment with ultrasound plus PpIX or ultrasound plus Au-PpIX the tumor was exposed to ultrasound 24 hr after PpIX or Au-PpIX administration (4). 

***Parameters for judgment on treatment efficacy***

The treatment effect was followed up via daily measurement of tumor diameters, including small diameter (a), large diameter (b) and tumor thickness (c) using a digital caliper with 0.01 mm precision, and estimation of tumor volume using equation (1) (16):

V= π/6 (a× b× c) (1)

The follow up of tumors were performed 70 days after treatment, which is the maximum survival of animals. For each tumor, the first day of treatment was considered as day zero and relative tumor volume in later days were accordingly normalized. On the basis of daily variations of the relative volume, doubling time and time being five times of the tumors was estimated in each group. The cumulative survival fraction and percentage of the lost tissue volume were also assessed in various groups.

***Statistical analysis***

All data were analyzed using SPSS 12 after performing normality test and selecting the proper comparative tests. According to the normality test of Kolmogorov-Smirnov, the data distribution was abnormal. Consequently, the Mann-Whitney test was used to compare relative tumor volume with a confidence level of 95%. Moreover, after calculating the cumulative survival fraction of animals via Kaplan-Meier method, Log Rank test was applied to compare them. Doubling time and time being five times of tumors was also compared in different groups using one- way ANOVA. 

*P<* 0.05 was considered to be significant. 

## Results

**Figure 4:**
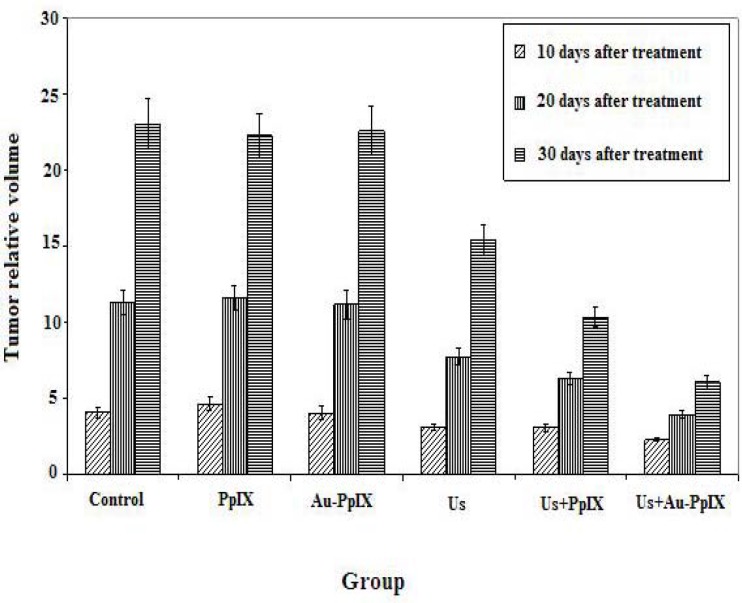
Mean ± SD of relative volume of tumors at 10, 20 and 30 days after treatment in the different groups (10 mice in each group). Control indicates tumors without treatment; PpIX, tumors treated with 5-mg/kg protoporphyrin IX alone; Au-PpIX, tumors treated with 5-mg/kg gold nanoparticle– protoporphyrin IX conjugate alone; US, tumors irradiated with ultrasound alone; US + PpIX, tumors irradiated with ultrasound 24 hr after injection of 5-mg/kg protoporphyrin IX; and US + Au-PpIX tumors irradiated with ultrasound 24 hr after injection of 5-mg/kg gold nanoparticle– protoporphyrin IX conjugate

**Figure 5:**
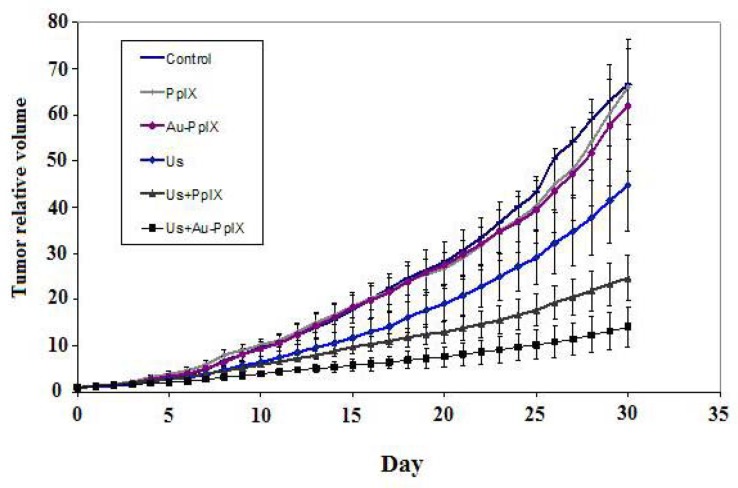
Mean ± SD of relative volume of tumors until 30 days post-treatment in the different groups (10 mice in each group). Control indicates tumors without treatment; PpIX, tumors treated with 5-mg/kg protoporphyrin IX alone; Au-PpIX, tumors treated with 5-mg/kg gold nanoparticle– protoporphyrin IX conjugate alone; US, tumors irradiated with ultra- sound alone; US + PpIX, tumors irradiated with ultrasound 24 hr after injection of 5-mg/kg protoporphyrin IX;

The effect of treatment protocols on the growth of the tumors volume in each group is compared in Figures 4 and 5. 

There are no inhibitory effects in control, PpIX and Au-PpIX groups. Ultrasound irradiation alone showed an insignificant antitumor effect which was enhanced by ultrasound plus PpIX (SDT). The synergistic inhibitory effect was significant when ultrasound plus Au-PpIX were used.

Statistical analysis shows that there is a significant difference in average relative volume of tumors in 10 days after treatment between ultrasound plus Au-PpIX received group and the other groups (*P<* 0.02), while there is no significant different between ultrasound plus Au-PpIX received group and the ultrasound received group (*P*= 0.20). Also there are significant differences in average relative volume of tumors in 20 and 30 days after treatment, between ultrasound plus Au-PpIX received group and the other groups (*P<* 0.005).

**Figure 6:**
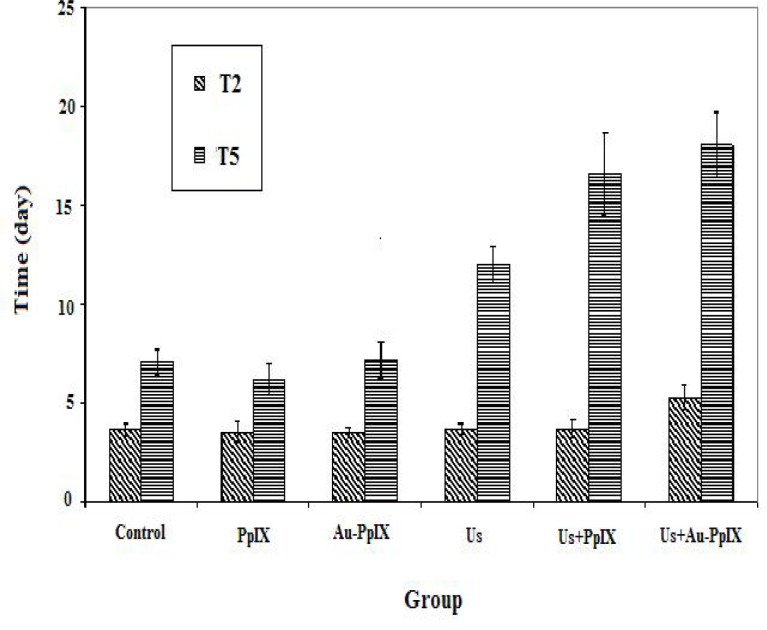
Mean±SD of doubling time and time being five times in the different groups (10 mice in each group). Control indicates tumors without treatment; PpIX, tumors treated with 5-mg/kg protoporphyrin IX alone; Au-PpIX, tumors treated with 5-mg/kg gold nanoparticle– protoporphyrin IX conjugate alone; US, tumors irradiated with ultra- sound alone; US + PpIX, tumors irradiated with ultrasound 24 hr after injection of 5-mg/kg protoporphyrin IX; and US + Au-PpIX tu- mors irradiated with ultrasound 24 hr after injection of 5-mg/kg gold nanoparticle–protoporphyrin IX conjugate.

Tumor doubling time and time being five times of different groups have been shown in Figures 6. 

The longest doubling time (T_2_) and time being five times (T_5_) are observed in ultrasound plus Au-PpIX, ultrasound plus PpIX and ultrasound received groups respectively.

The shortest T_2_ (3.67 days) belongs to control group and T_5_ (7.18 days) is obtained in Au-PpIX group.

**Figure 7:**
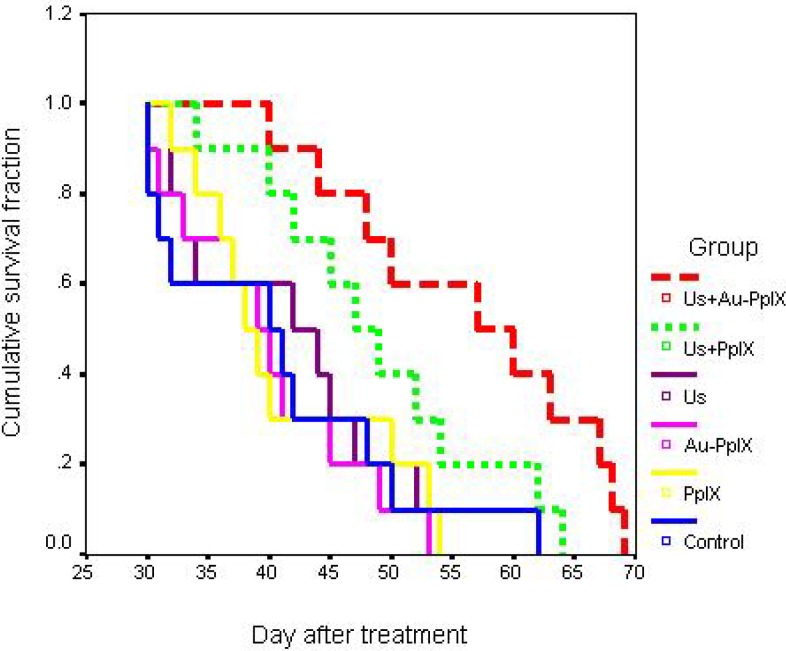
Cumulative survival fractions in the different groups (10 mice in each group). Control indicates tumors without treatment; PpIX, tumors treated with 5-mg/kg protoporphyrin IX alone; Au-PpIX, tumors treated with 5-mg/kg gold nanoparticle–protoporphyrin IX conjugate alone; US, tumors irradiated with ultrasound alone; US + PpIX, tumors irradiated with ultrasound 24 hr after injection of 5-mg/kg protoporphyrin IX; and US + Au-PpIX tumors irradiated with ultrasound 24 hr after injection of 5- mg/kg gold nanoparticle–protoporphyrin IX conjugate.

Statistical analysis showed that there is not a significant difference in doubling time between different groups (*P*> 0.06). But there is a significant difference in time being five times between therapeutic groups (ultrasound plus Au-PpIX or ultrasound plus PpIX) and each one of control, PpIX and Au-PpIX received groups (*P<* 0.004). It seems that both of these parameters can be a good indicator for an evaluation of tumor growth. Figure 7 shows cumulative survival fraction in various treatment groups.

In the ultrasound plus Au-PpIX group, mice showed a significant longer survival median (57.0 days) than control (40.0 days), PpIX (38.0 days), and Au-PpIX groups (39.0 days) (*P<* 0.01). But difference of the survival median between groups of the ultrasound plus Au-PpIX and ultrasound plus PpIX (SDT) was not significant (*P*= 0.07).

**Figure 8:**
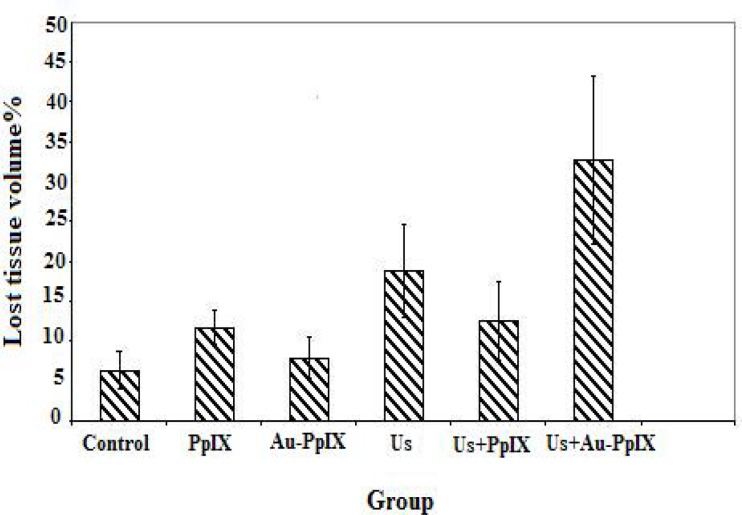
Percentages of lost tissue volume 24 hr after treatment in the different groups (5 tumors in each group). Control indicates tumors without treatment; PpIX, tumors treated with 5-mg/kg protoporphyrin IX alone; Au-PpIX, tumors treated with 5-mg/kg gold nanoparticle– protoporphyrin IX conjugate alone; US, tumors irradiated with ultrasound alone; US + PpIX, tumors irradiated with ultrasound 24 hr after injec- tion of 5-mg/kg protoporphyrin IX; and US + Au-PpIX tumors irradiated with ultrasound 24 hr after injection of 5-mg/kg gold nanoparticle– protoporphyrin IX conjugate.

Percentage of lost tissue volume (treated) in each group is shown in Figure 8. Based on the results, after 24 hr of treatment in ultrasound Plus Au-PpIX group, the lost tissue volume is in its largest amount (32.7%) and in control group contains the lowest amount (6.3%). 

## Discussion

The effects of ultrasound on biologic tissues have been studied extensively over the years. Ultrasound can destroy target tissue through various mechanisms, such as thermal and mechanical process (17). Another biological behavior of ultrasound is based on acoustic cavitation (2, 3) that utilizes a sonosensitizer as a mediator during sonodynamic therapy. As activation of sensitizing molecules is dependent to cavitation process, high intensity ultrasound is an important necessity. On the other hand, high intensity ultrasound can induces harmful effects on the healthy tissues which have surrounded tumor [8]. So still, the need for high intensity ultrasound is considered one of the existing challenges in SDT. Some researchers are interested in finding an appropriate approach to decrease intensity threshold of cavitation by using a good sonosensitizer. Up to now ATX-70 and PpIX are the best sonosensitizers introduced in SDT.

Tuziuti *et al* (9) in a research on correlation between the decrease in the cavitation threshold and the particles addition showed that the existence of particle into a liquid provides a nucleation site for cavitation bubble. This is due to its surface roughness. This fact, in its own turn, leads to the decrease in the cavitation threshold which is responsible for the increase in the number of bubbles, when the liquid is irradiated by ultrasound. Thus, in this context, one approach is based on providing the nucleation sites to be participating in the formation of cavities in order to reduce the threshold intensity of ultrasound needed for cavitation.

In this study, as a solution to reinforce the effect of ultrasound irradiation and treatment selectivity in the tumor, the use of a new designed sensitizer has been suggested.

GNPs are represented as a novel nano-material applied in the field of cancer therapy because of their special optical properties (10). In recent years, GNPs are also utilized in cancer diagnosis, treatment and drug delivery (18). Molecular bindings to the GNPs such as antibodies, carbohydrates and pharmacologic agents, are easy and feasible in order to target cancer cells. Non toxicity, good uptake by mammalian cells and antiangiogenesis property of GNPs is the most important aspects for its medical applications (18).

Prez *et al* (12) showed that the non-radiative relaxation time of PpIX in the presence of GNPs is longer than the similar time of PpIX without gold nanoparticles. This effect can be used in medical diagnostic and therapeutic applications. On the other hand, sonosensitivity and the capability of PpIX in imaging have been proven (12). Thus in this research, role of PpIX conjugated to GNPs in enhancing the efficiency of SDT was investigated.

Efficiency of each treatment on the tumors was evaluated through comparison of several viewpoints, including relative tumor volume 10, 20 and 30 days after treatment, the doubling time of the tumors, time being five times of the tumors, cumulative survival fraction and percentage of the lost tissue volume in each group.

There are no inhibitory effect in control, PpIX and Au-PpIX groups. Ultrasound irradiation alone showed an insignificant antitumor effect which was enhanced by ultrasound plus PpIX (SDT). The synergistic inhibitory effect was significant when ultrasound plus Au-PpIX were used.

Reduction in the relative volume of tumors after ultrasound treatment is noteworthy as compared to control, PpIX and Au-PpIX received groups. Since the water inside the cylindrical container between the probe and treated tumor did not show increased temperature, so the therapeutic effect of ultrasound waves cannot be related to the effect of hyperthermia. This effect seems to be caused by mechanical shear forces and cavitation.

Significant reduction in the relative volume of tumors after 30 days, in the group receiving ultrasound and PpIX (SDT) in comparison with control group, and each one of groups that has received PpIX, Au-PpIX and ultrasound alone, confirms sono-activation of the sensitizers (SDT). 

The best response to treatment appears in the received ultrasound plus Au-PpIX group. According to these results, the best response to the treatment has been observed in ultrasound plus Au-PpIX group and in this therapeutic group, and relative shrinking of tumor volume has been higher than in other groups.

As results of pathology show, the largest lost tissue volume is related to the ultrasound plus Au-PpIX group. This result indicates that tumor's response to treatment occurs 24 hr after treatment. The lost tissue volume in sonodynamic treatment was minimal. This effect seems to be necessary for the treatment of this tumor.

As the cytotoxicity of sonosensitization in biological tissues is intermediate by single oxygen (14), the latter finding can be related to several courses of action:

1. Existence of PpIX as a sonosensitizer and GNPs as cavitation nuclei. In other words, nanoparticles might be able to act as sites for cavitation and have increased cavitation rate. 

2. Entrance of PpIX to the tumor cells has probably been faciliated by GNPs. 

3. Increased collapsing of cavities is another feasible process. 

However, the free radical molecules produced by collapsing of cavities are in high energy states, and this energy could be transfered to the PpIX molecules to form the excited states PpIX. The transferred energy from activated PpIX to oxygen molecule produces singlet oxygen (14).

## Conclusion

The results of the present investigation suggest that Au-PpIX has a better potential than PpIX as sonosensitizer in order to treat CT26 tumors by SDT. Experimental investigation showed that the synergistic inhibitory effect is significant when ultrasound plus Au-PpIX were used. This finding can be related to more uptake of PpIX by the tumor cells, presence of GNPs as cavitation nuclei or more collapsing of cavities. 
